# The Role of Global Longitudinal Strain in Subclinical Hypothyroid Patients With Heart Failure

**DOI:** 10.7759/cureus.46973

**Published:** 2023-10-13

**Authors:** Nismat Javed, Vibha Hayagreev, Angel DeLaCruz, Muhammad Saad, Amandeep Singh, Timothy Vittorio

**Affiliations:** 1 Internal Medicine, BronxCare Health System, Bronx, USA; 2 Cardiology, BronxCare Health System, Bronx, USA; 3 Cardiovascular Medicine, BronxCare Health System, Bronx, USA; 4 Cardiovascular Disease, BronxCare Health System, Bronx, USA

**Keywords:** tte, ste, global longitudinal strain, subclinical hypothyroidism, heart failure

## Abstract

Introduction

There is considerable evidence to suggest the role of thyroid hormone in acute coronary syndrome (ACS), but less is known about its prognostic role in heart failure (HF). We aimed to assess the role of global longitudinal strain (GLS) in patients hospitalized with HF and underlying subclinical hypothyroidism (SCHS).

Methods

A retrospective analysis of 161 subjects was conducted by dichotomizing them into HF with preserved ejection fraction (HFpEF) and HF with reduced ejection fraction (HFrEF) subgroups. SCHS was defined as a thyroid stimulating hormone level >4.50 mIU/L with a normal thyroxine level based on the evaluation of limits for lab markers from prior studies. HFpEF and HFrEF were defined as left ventricular ejection fraction (LVEF)>40% and LVEF≤40%, respectively, based on American College of Cardiology (ACC) guidelines. An abnormal speckled transthoracic echocardiographic (TTE) strain was defined as a left ventricular global longitudinal strain (LVGLS) value of >-15%.

Results

The mean age of the population was 62±8 years, and 55% were female. LVGLS was present in 121 patients with underlying SCHS (p<0.05). The patients with SCHS and abnormal LVGLS were found to have deranged left ventricular echocardiographic parameters (p<0.05). The derangements were greater for SCHS patients with HFrEF and abnormal LVGLS (p<0.05). Readmission rates at 30 days and in-hospital mortality were higher in SCHS patients with abnormal LVGLS (p<0.05).

Conclusion

The SCHS is associated with abnormal GLS in HF patients (either HFpEF or HFrEF) that results in remodeling and adverse outcomes, including mortality and readmission rates. Further studies are warranted to validate these findings in a larger population data pool.

## Introduction

The prevalence of subclinical hypothyroidism (SCHS) depends on many varying factors, including age, sex, race, region, and method of thyroid stimulating hormone (TSH) measurement [1]. Estimates of global prevalence vary from 0.9% in China to 18.0% in Northern Europe [1]. SCHS is more commonly found in areas with an iodine-sufficient diet [1]. The prevalence of SCHS in the United States ranges from 4.3% to 9.4% [2]. In contrast, heart failure (HF) affects a larger population worldwide-approximately 64 million people worldwide [3]. The American Heart Association (AHA) reported that between 2013 and 2016, 6.2 million people had HF in the United States. The AHA estimates for 2021 revealed the prevalence of HF to be 6 million, or approximately 1.8% of the total United States population [4]. The disease burden is expected to increase by 46% by 2030 [5]. The SCHS can affect cardiovascular physiology through various mechanisms, including accelerated atherosclerosis via increased transcription of smooth muscle cells, alteration of lipid levels, and changes in endothelial activity [6-8]. Additionally, cardiac function can be affected by thyroid imbalance, which might lead to the progression of HF [9].

Diastolic dysfunction has been observed in patients with SCHS. In particular, a higher E/E′ ratio (peak velocity of blood flow across the mitral valve in early diastole/mitral annular velocity in early diastole ratio) was observed in patients with SCHS [7]. Endothelial dysfunction derived from apototic microparticles, alteration of nitric oxide (NO) production, and increased inflammatory markers are also associated with SCHS [9]. Patients with SCHS develop left ventricular dysfunction at an earlier stage [10]. SCHS is associated with reduced left ventricular global longitudinal strain (LVGLS), which is one of the predictors for HF [11,12]. However, the utility of LVGLS for the long-term outcomes of patients with SCHS and HF remains limited.

With the increasing burden of HF, it is imperative to further explore the characteristics of patients with LVGLS associated with underlying SCHS and HF. The aim of the study was to determine demographic, clinical characteristics and cardiovascular outcomes in patients to assess the role of GLS in patients hospitalized for HF and with underlying SCHS.

## Materials and methods

Patient selection

A retrospective review of in-hospital admissions from December 31st, 2006, to December 31st, 2021, was performed at our hospital. Data pertaining to hospitalizations with HF as the primary or secondary diagnosis was utilized. The International Classification of Diseases, Ninth Revision, Clinical Modification (ICD-9-CM) and ICD-10 were used to identify HF hospitalizations older than 18 years of age [13]. The codes utilized in the study are summarized in Table 1.

**Table 1:** ICD codes used

ICD-9	244.9, 428.0, 428.1, 428.2, 428.20, 428.21, 428.22, 428.23, 428.3, 428.3, 428.31, 428.32, 428.33, 428.4, 428.40, 428.41, 428.42, 428.43, 428.9
ICD-10	E02, E03, I50, I50.1, I50.2, I50.20, I50.21, I50.22, I50.23, I50.3, I50.30, I50.31, I50.32, I50.33, I50.4, I50.40, I50.41, I50.42, I50.43, I50.8, I50.81, I50.810, I50.811, I50.812, I50.813, I50.814, I50.82, I50.83, I50.84, I50.89, I50.9

The records were reviewed to ascertain the purpose of admission, which was treatment of HF and SCHS. When patients were hospitalized more than once during the study period, only data from the first hospitalization was included for analysis. Patients who were under 18 years of age, pregnant, or transferred to our institution from another hospital were excluded. Hospitalizations with HF and SCHS were then identified from the main cohort. HF hospitalizations were stratified into HF with preserved ejection fraction (HFpEF) and HF with reduced ejection fraction (HFrEF). HFpEF was defined as left ventricular ejection fraction (LVEF >40%), and HFrEF was defined as LVEF≤40% as per ACC guidelines. SCHS was defined as a TSH level of >4.5 mIU/L with a normal thyroxine level based on cutoffs used in previous clinical studies with a similar cohort. LVGLS was determined in all cohorts. An abnormal transthoracic echocardiographic (TTE) strain was defined as LVGLS>-15%. Patients were then categorized into cohorts with normal and abnormal LV GLS.

Protocol followed for transthoracic echocardiography

The guidelines from the American Society of Echocardiography were followed for two-dimensional transthoracic echocardiography (2D-TTE) and speckle tracking echocardiography (STE) [14]. Images from apical four-chamber, two-chamber, and three-chamber views were used for the measurement of LVGLS. The gray-scale frame rate was optimized to ensure satisfactory tracking and prevent compromising the spatial resolution. The images were then analyzed. The apical long-axis image was analyzed first. A region of interest, including the entire myocardial thickness, was generated with segmental division and the calculation of values for GLS. The apical four-chamber and two-chamber images were evaluated in a similar manner to obtain an average value for LVGLS.

Statistical analysis

Data about age, gender, cardiovascular outcomes, and echocardiographic parameters were extracted. Statistical analyses were performed using IBM Corp. Released 2012. IBM SPSS Statistics for Windows, Version 21.0. Armonk, NY: IBM Corp. Continuous variables were expressed as mean with standard deviations (±SD) or median with interquartile range (±IQR), depending on the data distribution. Categorical data were expressed as frequency with percentages. Intergroup and intragroup comparisons of continuous variables were computed using the T-test, and categorical variables were evaluated using the chi-square test or analysis of variance (ANOVA). All p-values were two-sided with a significance limit of <0.05. A multivariable logistic regression analysis was performed with variables contributing to hospitalizations in all cohorts.

## Results

The total number of patients included in the study was 161. The mean age of the study population was 62±8 years. There were 72 males and 89 females in the study (p>0.05). LVGLS was present in 121 patients with underlying SCHS (p<0.05).

Comparison of outcomes in subclinically hypothyroid patients with abnormal LVGLS and normal LVGLS

The TTE characteristics for these patients are shown in Table 2. 

**Table 2:** TTE parameters in patients with normal and abnormal LVGLS with underlying SCHS LVGLS: Left Ventricular Global Longitudinal Strain, RVSP: Right Ventricular Systolic Pressure, LVEDD: Left Ventricular End Diastolic Diameter, LVEDV: Left Ventricular End Diastolic Volume, LVESV: Left Ventricular End Systolic Volume, LVESD: Left Ventricular End Systolic Diameter, LVSD: Left Ventricular Systolic Diameter, LVPW: Left Ventricle Posterior Wall Diameter, LVMI: Left Ventricular Mass Index, LA: Left Atrium, LAVI: Left Atrium Volume Index, TR: Tricuspid Regurgitation, Peak E: Peak flow velocity in left ventricular relaxation in early diastole, Peak A: Peak velocity flow in late diastole caused by atrial contraction, E/A ratio of peak E to peak A velocity, RWT: Relative wall thickness, SD: Standard deviation, TTE: Transthoracic echocardiogram A p-value of <0.05 was considered significant.

Variable	Abnormal LVGLS	Normal LVGLS	p-value
RVSP in mmHg (Mean±SD)	49.30±20.15	44.74±22.35	0.175
LVEDD in cm (Mean±SD)	6.04±3.04	5.50±1.42	0.200
LVEDV in ml (Mean±SD)	128.58±76.38	112.83±83.64	0.214
LVESV in ml (Mean±SD)	90.79±67.07	77.23±72.57	0.221
LVESD in cm (Mean±SD)	4.72±1.47	4.15±1.89	0.028
LVSD in cm (Mean±SD)	1.12±0.27	1.00±0.37	0.017
LVPW in cm (Mean±SD)	1.13±0.27	1.01±0.37	0.013
LVMI in g/m^2^(Mean±SD)	138.65±45.56	111.82±56.99	0.001
LA Diameter in cm (Mean±SD)	4.54±4.11	4.16±1.89	0.505
LAVI in m^2^(Mean±SD)	26.30±29.02	20.24±30.39	0.200
TR Gradient in mmHg (Mean±SD)	38.21±16.59	32.48±20.11	0.046
TR Velocity in m/s (Mean±SD)	3.87±9.79	4.44±14.56	0.760
Peak E in m/s (Mean±SD)	0.85±0.46	0.79±0.55	0.490
Peak A in m/s (Mean±SD)	0.51±0.49	0.43±0.44	0.283
E/A ratio (Mean±SD)	1.38±1.16	1.10±1.15	0.118
Deceleration time in msec (Mean±SD)	110.15±102.94	99.29±118.77	0.532
RWT in cm (Mean±SD)	0.41±0.16	0.37±0.16	0.097

Patients with SCHS and abnormal LVGLS had significantly greater LVESD, LVSD, LVPW, LVMI, and a higher tricuspid regurgitant gradient (p<0.05). Table 3 discusses cardiovascular outcomes in a similar fashion. 

**Table 3:** Cardiovascular outcomes in patients with normal and abnormal LVGLS with underlying SCHS LVGLS: Left ventricular global longitudinal strain, N: Frequency, %: Percentage A p-value of <0.05 was considered significant.

Variable (N, %)	Abnormal LVGLS (N, %)	Normal LVGLS (N, %)	p-value
Shock requiring inotropic support	55 (79.7)	14 (20.3)	0.276
Shock requiring vasopressor support	55 (76.4)	17 (23.6)	0.130
ICU Admissions	46 (68.7)	21 (31.3)	0.534
Readmission at 30 days	65 (70.7)	27 (29.3)	0.821
Readmission at 90 days	48 (59.3)	33 (40.7)	0.734
Readmission at 180 days	43 (55.1)	35 (44.9)	0.642

Although patients with both abnormal LVGLS and SCHS were more likely to require ICU level of care (46/67, 68.7%), this association was not significant. Similarly, inotropic support (55/69, 79.7%) and vasopressor support (55/72, 76.4%) were more common in patients with SCHS and abnormal LVGLS; however, this association was not significant. Additionally, higher readmission rates were observed in the same cohort, but it was not significant.

Comparison of outcomes in subclinically hypothyroid patients with abnormal LVGLS and normal LVGLS stratified by HF subtype

The TTE parameters were analyzed in all HF cohorts, as shown in Table 4. 

**Table 4:** TTE parameters in all cohorts LVGLS: Left ventricular global longitudinal strain, HFrEF: Heart failure with reduced ejection fraction, HFpEF: Heart failure with preserved ejection fraction, RVSP: Right ventricular systolic pressure, LVEDD: Left ventricular end diastolic diameter, LVEDV: Left ventricular end diastolic volume, LVESV: Left ventricular end systolic volume, LVESD: Left ventricular end systolic diameter, LVSD: Left ventricular systolic diameter, LVPW: Left ventricle posterior wall diameter, LVMI: Left ventricular mass index, LA: Left atrium, LAVI: Left atrium volume index, TR: Tricuspid regurgitation, Peak E: Peak flow velocity in left ventricular relaxation in early diastole, Peak A: Peak velocity flow in late diastole caused by atrial contraction, E/A: Ratio of peak E to peak A velocity, RWT: Relative wall thickness, SD: Standard deviation, TTE: Transthoracic echocardiogram A p-value of < 0.05 was considered significant.

Variable	Abnormal LVGLS			Normal LVGLS		
	HFrEF	HFpEF	p-value	HFrEF	HFpEF	p-value
RVSP in mmHg (Mean±SD)	49.73±19.77	43.47±25.65	0.612	47.17±22.61	35.23±19.23	0.226
LVEDD in cm (Mean±SD)	6.08±3.13	5.49±1.04	0.459	5.55±1.53	5.30±0.92	0.160
LVEDV in ml (Mean±SD)	131.48±77.61	89.34±43.5	0.551	117.04±88.62	96.35±30.59	0.249
LVESV in ml (Mean±SD)	93.17±67.79	58.63±48.78	0.417	82.56±75.14	56.42±49.36	0.239
LVESD in cm (Mean±SD)	4.76±1.46	4.26±1.70	0.001	4.27±1.97	3.68±1.54	0.042
LVSD in cm (Mean±SD)	1.12±0.28	1.20±0.21	0.001	0.97±0.41	1.14±0.14	0.030
LVPW in cm (Mean±SD)	1.12±0.25	1.13±0.09	0.001	0.97±0.39	1.14±0.09	0.036
LVMI in g/m^2 ^(Mean±SD)	138.44±45.27	141.50±52.69	0.001	110.90±59.96	115.42±45.58	0.007
LA Diameter in cm (Mean±SD)	4.54±4.26	4.54±0.56	0.346	4.13±1.86	4.31±0.78	0.462
LAVI in g/m^2 ^(Mean±SD)	24.64±8.83	48.75±22.45	0.798	23.60±2.43	7.08±5.28	0.190
TR Gradient in mmHg (Mean±SD)	37.89±16.87	42.43±12.20	0.881	31.37±20.56	36.86±18.39	0.076
TR Velocity in m/s (Mean±SD)	3.93±0.15	3.21±0.48	0.734	4.85±1.63	2.86±1.09	0.820
Peak E in m/s (Mean±SD)	0.84±0.47	0.96±0.37	0.981	0.76±0.55	0.95±0.52	0.507
Peak A in m/s (Mean±SD)	0.50±0.15	0.66±0.18	0.118	0.37±0.30	0.64±0.57	0.262
E/A ratio (Mean±SD)	1.37±1.18	1.66±0.86	0.265	1.13±0.18	0.97±0.06	0.117
Deceleration time in msec (Mean±SD)	105.65±101.36	170.90±112.05	0.274	95.59±18.44	113.82±24.24	0.556
RWT in cm (Mean±SD)	0.41±0.16	0.42±0.09	0.266	0.35±0.17	0.44±0.09	0.104

SCHS patients with abnormal LVGLS and HFrEF had significantly larger LVESD, LVSD, LVPW, and LVMI compared to patients with abnormal LVGLS and HFpEF (p<0.05). Similar results were observed in HF patients with normal LVGLS (p<0.05). Table 5 discusses cardiovascular outcomes in the same cohorts. 

**Table 5:** Cardiovascular outcomes in all cohorts LVGLS: Left ventricular global longitudinal strain, HFrEF: Heart failure with reduced ejection fraction, HFpEF: Heart failure with preserved ejection fraction, N: Frequency, %: Percentage A p-value of <0.05 was considered significant.

Variable (N, %)	Abnormal LVGLS (N, %)				Normal LVGLS (N, %)			
	HFrEF	HFpEF	p-value	HFrEF		HFpEF	p-value	
Shock requiring inotropic support	14 (77.8)	0 (0.0)	0.277	4 (22.2)		0 (0.0)	0.295	
Shock requiring vasopressor support	17 (80.9)	0 (0.0)	0.244	4 (19.1)		0 (0.0)	0.295	
ICU admissions	20 (58.8)	1 (2.9)	0.670	11 (32.4)		2 (5.9)	0.615	
Readmission at 30 days	64 (65.9)	1 (1.1)	0.010	26 (26.8)		6 (6.2)	0.741	
Readmission at 90 days	46 (62.1)	2 (2.7)	0.330	23 (31.1)		3 (4.1)	0.136	
Readmission at 180 days	41 (61.1)	2 (2.9)	0.464	19 (28.5)		5 (7.5)	0.938	

Readmission at 30 days was found to be significantly higher in SCHS patients with both abnormal GLS and HFrEF (64/97, 65.9%, p<0.05). However, the observations in the other cohorts were not significant. A multivariate logistic regression model was used to determine predictors for dependent variables, including inotrope support, vasopressor support, ICU admissions, and readmission rates with abnormal LVGLS due to underlying SCHS in all HF patients, as shown in Table 6. The model was adjusted for age, gender, and baseline TTE parameters, for example, LVEF, systolic, and diastolic diameters at admission. 

**Table 6:** Multivariate regression analysis A p-value of <0.05 was considered significant.

Variable	Adjusted Odds Ratio (aOR)	95% Confidence Interval [Lower Bound-Upper Bound]	p-value
Shock requiring inotropic support	0.511	0.123-2.120	0.355
Shock requiring vasopressor support	0.429	0.105-1.748	0.238
ICU Admissions	1.542	0.605-3.927	0.364
Readmission at 30 days	1.708	0.767-3.801	0.190
Readmission at 90 days	1.792	0.818-3.924	0.145
Readmission at 180 days	1.747	0.803-1.798	0.159

Mortality analysis in subclinical hypothyroid patients with abnormal and normal LVGLS

**Figure 1:**
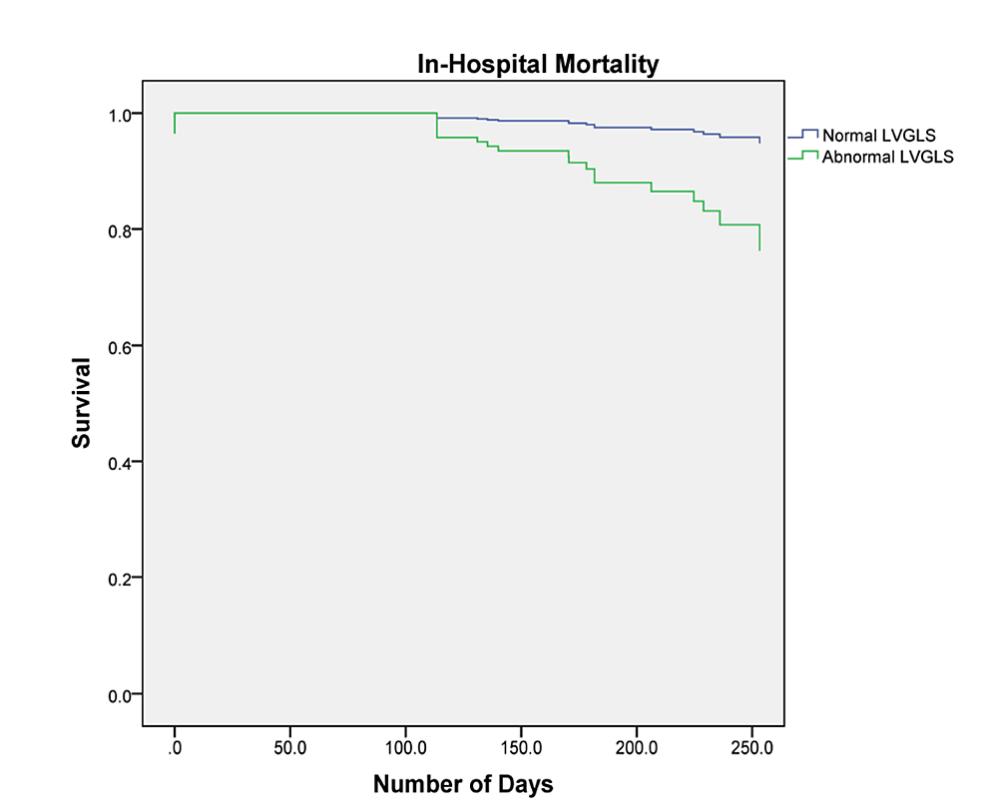
In-hospital mortality LVGLS: Left ventricular global longitudinal strain

The Cox-regression results are illustrated in Figure 1. 

SCHS patients with abnormal LVGLS had significantly lower survival times compared to patients with normal LVGLS (p<0.05). The results were similar in all HF categories (p<0.05).

## Discussion

In our study, SCHS was significantly associated with abnormal LVGLS that was crucial to remodeling, function, and outcomes in HF patients. The current study demonstrated for the first time that SCHS individuals with abnormal LVGLS had significantly larger LVESD (4.72±1.47), LVSD (1.12±0.27), LVPW (1.13±0.27), LVMI (138.65±45.56), and TR gradients (38.21±16.59) compared to SCHS patients with normal LVGLS. In our study, patients with SCHS and abnormal LVGLS had a higher 30-day readmission rate (64/97, 65.9%, p<0.05) and in-hospital mortality. 

Many studies have discussed the role of SCHS in LV and LA dynamics [15-17]. SCHS alters the hemodynamics of the LV in multiple ways. Thyroid hormones accelerate cardiac contractility through both direct stimulation and increased peripheral oxygen turnover [18]. At the cellular level, these hormones also have an impact on intracellular calcium handling mediated by second-messenger systems [19]. The entire cycle of events alters myocardial fiber orientation and causes collagen changes that could lead to left ventricle hypertrophy (LVH) [20-23]. The SCHS is associated with increased systemic vascular resistance and decreased cardiac output, further impairing the mechanics involved [20,24]. Additional cardiovascular risk factors associated with SCHS could attenuate cardiac remodeling [25]. Although LA mechanics, particularly LA reservoir, have been discussed as a more independent predictor of HF [17], abnormal LVGLS has also been discussed in smaller studies [16,23].

The higher values of LVESD, LVSD, LVPW, and LVMI suggest that the SCHS primarily results in diastolic dysfunction, as discussed in prior studies [20,21]. Additionally, it is important to note that the parameters were significantly increased in patients with HFrEF, suggesting that the remodeling process is more pronounced in patients with existing systolic dysfunction. LV systolic dysfunction has been studied in patients with LVEF<35% previously [26]. Increased LV systolic dysfunction was associated with the irreversibility of cardiac remodeling and a reduced likelihood of LVEF recovery [26]. However, these findings are known to be independent risk factors for HF and subsequent deterioration as opposed to being associated with LVGLS [27,28].

LV geometry, specifically as shown by LVGLS in SCHS patients, has a more complex role to play in both cohorts, leading to varied outcomes. In this regard, strain patterns can induce overlapping dysfunction through three possible mechanisms. Prevalent longitudinal dysfunction, as measured by longitudinal strain, would initially manifest as subendocardial dysfunction compensated by parietal hypertrophy and the contractile reserve of the epicardial layer. In this case, both systolic and diastolic decompensations might coexist. Transmural dysfunction, as measured by circumferential strain, might lead to dysfunction typical of HFrEF [29].

Abnormal LVGLS has been discussed as an indicator for adverse outcomes in other cohorts of patients, including coronary artery disease and cardiac transplant [30,31]. Greater rates of morbidity and mortality have been discussed in HF patients with LVGLS [32]. Risk stratification models have been described in relation to LVGLS in HF patients [33]. LVGLS is more reflective of myocardial motion as compared to LVEF [33]. However, association with underlying SCHS has not been assessed. Myocardial motion would be hampered by the vicious cycle of remodeling because of thyroid-induced changes that could result in long-term adverse outcomes.

Limitations

The study has a few limitations. The analysis was retrospective in nature, with a relatively smaller sample size. Data about right atrial and right ventricular dynamics were limited. Additionally, data regarding circumferential strain was scarce.

## Conclusions

SCHS patients with abnormal LVGLS have higher and larger LVESD, LVSD, LVPW, LVMI, and TR gradients correlating with diastolic and systolic remodeling, which can increase the risk of mortality. Additionally, these deranged parameters can also lead to an increase in readmission rates, specifically the 30-day readmission rate. However, it must be highlighted that the higher readmission rate could be due to non-adherence to guideline-directed medical therapy. Additional large-scale studies are warranted to determine the impact of these measures on a national scale in order to streamline guidelines for diagnosis, management, and prognosis.
